# RNase III and RNase E Influence Posttranscriptional Regulatory Networks Involved in Virulence Factor Production, Metabolism, and Regulatory RNA Processing in Bordetella pertussis

**DOI:** 10.1128/mSphere.00650-21

**Published:** 2021-08-18

**Authors:** Gyles Ifill, Travis Blimkie, Amy Huei-Yi Lee, George A. Mackie, Qing Chen, Scott Stibitz, Robert E. W. Hancock, Rachel C. Fernandez

**Affiliations:** a Department of Microbiology and Immunology, University of British Columbia, Vancouver, British Columbia, Canada; b Department of Biochemistry and Molecular Biology, University of British Columbia, Vancouver, British Columbia, Canada; c Division of Bacterial, Parasitic, and Allergenic Products, Center for Biologics Evaluation and Research, Food and Drug Administration, Silver Spring, Maryland, USA; University of Iowa

**Keywords:** *Bordetella pertussis*, RNA processing, RNase E, RNase III, posttranscriptional regulation, regulatory networks, virulence regulation

## Abstract

Bordetella pertussis has been shown to encode regulatory RNAs, yet the posttranscriptional regulatory circuits on which they act remain to be fully elucidated. We generated mutants lacking the endonucleases RNase III and RNase E and assessed their individual impact on the B. pertussis transcriptome. Transcriptome sequencing (RNA-Seq) analysis showed differential expression of ∼25% of the B. pertussis transcriptome in each mutant, with only 28% overlap between data sets. Both endonucleases exhibited substantial impact on genes involved in amino acid uptake (e.g., ABC transporters) and in virulence (e.g., the type III secretion system and the autotransporters *vag8*, *tcfA*, and *brkA*). Interestingly, mutations in RNase III and RNase E drove the stability of many transcripts, including those involved in virulence, in opposite directions, a result that was validated by qPCR and immunoblotting for *tcfA* and *brkA*. Of note, whereas similar mutations to RNase E in Escherichia coli have subtle effects on transcript stability, a striking >20-fold reduction in four gene transcripts, including *tcfA* and *vag8*, was observed in B. pertussis. We further compared our data set to the regulon controlled by the RNA chaperone Hfq to identify B. pertussis loci influenced by regulatory RNAs. This analysis identified ∼120 genes and 19 operons potentially regulated at the posttranscriptional level. Thus, our findings revealed how changes in RNase III- and RNase E-mediated RNA turnover influence pathways associated with virulence and cellular homeostasis. Moreover, we highlighted loci potentially influenced by regulatory RNAs, providing insights into the posttranscriptional regulatory networks involved in fine-tuning B. pertussis gene expression.

**IMPORTANCE** Noncoding, regulatory RNAs in bacterial pathogens are critical components required for rapid changes in gene expression profiles. However, little is known about the role of regulatory RNAs in the growth and pathogenesis of Bordetella pertussis. To address this, mutants separately lacking ribonucleases central to regulatory RNA processing, RNase III and RNase E, were analyzed by RNA-Seq. Here, we detail the first transcriptomic analysis of the impact of altered RNA degradation in B. pertussis. Each mutant showed approximately 1,000 differentially expressed genes, with significant changes in the expression of pathways associated with metabolism, bacterial secretion, and virulence factor production. Our analysis suggests an important role for these ribonucleases during host colonization and provides insights into the breadth of posttranscriptional regulation in B. pertussis, further informing our understanding of B. pertussis pathogenesis.

## INTRODUCTION

Bacterial pathogens must be able to rapidly adapt to the range of environments encountered within the host during infection. Successful colonization requires tightly regulated gene expression balancing stress state responses, resisting specific immune and nutritional challenges, and production of virulence factors (1, 2). Many environmental signals are detected by sensor kinases that coordinate rapid transcriptional changes through the phosphorylation of response regulators (3). However, rapid changes in gene expression can also be coordinated at the posttranscriptional level by regulatory RNAs. In recent years, there has been substantial expansion in the number of identified regulatory RNAs and in the understanding of the wide range of adaptive responses in which they are involved. Regulatory RNAs have been shown to play roles in modulating quorum-sensing responses, changing cell surface structures, and fine-tuning bacterial metabolism (2, 4, 5). There are three main classes into which regulatory RNAs are grouped: (i) regulatory elements found in the 5′ untranslated region (UTR), such as riboswitches and RNA thermometers; (ii) *cis*-encoded, antisense RNAs (asRNA) that are transcribed from the opposite strand of the target mRNA; and (iii) small noncoding regulatory RNAs (sRNA), 50- to 400-nucleotide *trans*-encoded transcripts that require an RNA chaperone, such as Hfq, to catalyze interactions with the target transcript (6). Interactions between regulatory RNAs and the mRNA targets often lead to changes in the secondary structure of the target mRNA. These changes can modulate rates of translation by altering accessibility to ribosome binding sites or leading to cleavage of the target mRNAs. Cleavage events can result in either the degradation or stabilization of the target mRNA, whereby the regulatory RNAs are associated with directing the activity of one or several ribonucleases that are involved in cleavage of their target RNA(s) (7).

Ribonucleases involved in regulatory RNA turnover can digest from the terminal ends of an RNA transcript (exonucleases) or cleave at an internal site (endonucleases) (8). Two key endonucleases involved in regulatory RNA processing in bacterial pathogens are RNase III and RNase E. RNase III is an endoribonuclease with specific activity for double-stranded RNA (dsRNA). It acts as a homodimer with a key role in the maturation of ribosomal RNAs (rRNAs) and transfer RNAs (tRNAs) (9, 10). RNase III has been shown to influence Staphylococcus aureus virulence through cleavage of the RNAIII transcript (11, 12) and has an important role in mediating changes of metabolic state in Escherichia coli (9). Due to its specificity for double-stranded RNA, RNase III has also been implicated in processing asRNAs and their targets (13).

RNase E is an essential endoribonuclease that forms a homotetramer associated with the bacterial inner membrane. The N-terminal RNase domain containing the catalytic site is required for the maturation of rRNAs but is also involved in the degradation of many cellular transcripts. Three other proteins (RhlB, enolase, and PNPase) complex with RNase E through its C-terminal half, forming a multicomponent structure known as the degradosome (8, 14). The C-terminal half has been shown to be required for interactions with the RNA chaperone Hfq, with the sRNA and mRNA molecules bound to the proximal and distal faces of Hfq, respectively (15, 16). Through these interactions with Hfq and its role in sRNA turnover, RNase E has been shown to regulate the expression of virulence factors and mediate several stress responses at the posttranscriptional level (17–19).

In Bordetella pertussis, the causative agent of whooping cough, virulence factor production is centrally controlled by the BvgAS two-component system (20). The system coordinates two main transcriptional states known as the Bvg+ and Bvg− modes. When the cells are grown at 35 to 37°C, B. pertussis enters the Bvg+ phase in which the sensor kinase BvgS phosphorylates the response regulator BvgA. BvgA-P is then able to promote the expression of virulence-associated genes (*vags*) and suppress the expression of virulence-repressed genes (*vrgs*) (20–22). BvgS can be modulated by lowering the growth temperature or by adding millimolar concentrations of nicotinic acid or MgSO_4_ to growth media *in vitro*. This Bvg− mode is further characterized by the repression of *vags* and the expression of *vrgs*. Finally, a third gene expression profile can be produced by the BvgAS system. Known as the Bvg intermediate mode (Bvg^i^), it is characterized by the expression of *vrgs*, *vags* not requiring full BvgA-P activation, and Bvg^i^-phase-specific polypeptides (Bips), of which BipA has been the most widely studied (23, 24).

Indeed, the regulation of genes within the virulence regulon has many complexities, since several overlapping regulatory systems have been shown to modulate the expression of genes in the Bvg+ or Bvg− modes (25, 26). Interestingly, Hfq has been shown to be required for the expression of many virulence factors (27). Additionally, recent studies have also identified potential virulence-associated sRNAs, suggesting the involvement of regulatory RNAs in pathogenesis (27–30).

Even though regulatory RNAs have been identified, there are few examples in B. pertussis in which the targets for these regulatory RNAs have been identified. To understand more about the contribution of regulatory RNAs to posttranscriptional gene regulation in *Bordetella*, we generated mutant alleles of both the endonucleases RNase III and RNase E and analyzed their impact on the B. pertussis global transcriptome using RNA-Seq. Here, we show that both endonucleases are involved in processing approximately 25% of the B. pertussis transcriptome, including transcripts associated with bacterial metabolism and virulence factor production. In this regard, we further examined two genes encoding virulence factors which are representative of both the subtle and more dramatic differential expression patterns observed within our data set; *brkA* and *tcfA* encode autotransporter proteins involved in serum resistance and host colonization, respectively (31, 32). Validating the robustness of the RNA-Seq analysis, we showed that changes in transcript abundance for both autotransporters is mirrored at the protein level. Through comparisons of our RNA-Seq data set to published studies (28, 30), we determined more than 100 gene loci that are potentially influenced by regulatory RNAs. This study thus probes the posttranscriptional landscape of B. pertussis in showing how altered RNA processing impacts the B. pertussis transcriptome, while also identifying gene loci potentially influenced by asRNA and sRNA.

## RESULTS

### Generation and growth characteristics of the RNase III mutant, BPrncD45A.

*Cis*-encoded asRNAs are transcribed from the opposite strand from the target RNA and can interact with the target through complementary base pairing (6). Since RNase III specifically cleaves RNA-RNA duplexes, it has been implicated in several asRNA control mechanisms. In B. pertussis, the *rnc* gene encoding RNase III is part of a five-gene operon consisting of *lepA*, *lep*, *rnc*, *era*, and *recO* (33). Both *lep* and *era* have been identified as essential genes in B. pertussis (34) and overlap with the start and stop codon of the *rnc* gene, respectively. Repeated attempts to delete *rnc* were unsuccessful, likely due to potential polar effects. Previous *in vitro* studies of Escherichia coli RNase III have shown that mutations at the active site aspartic acid (D45) resulted in ⁓30,000-fold reduction in RNase activity (35). Also, approaches in Streptomyces coelicolor to diminish RNase III nuclease activity while maintaining other important biological functions also targeted the active site aspartic acid (36). Furthermore, the importance of this residue in endonuclease activity was also modeled using the structure of RNase III from Aquifex aeolicus (37). Amino acid sequences of the *rnc* genes from B. pertussis, E. coli, S. coelicolor, and *A. aeolicus* were aligned (Fig. S1A in the supplemental material) and residues in B. pertussis RNase III corresponding to known active site motifs were mapped. As shown in Fig. S1A, the D45 residue shown to be important for catalytic activity (35–37) is highly conserved in B. pertussis. We thus chose to substitute this aspartic residue for alanine on the chromosomal copy of the *rnc* gene using allelic exchange (38) and assessed the impact of this mutation on asRNA regulation in B. pertussis.

**FIG 1:**
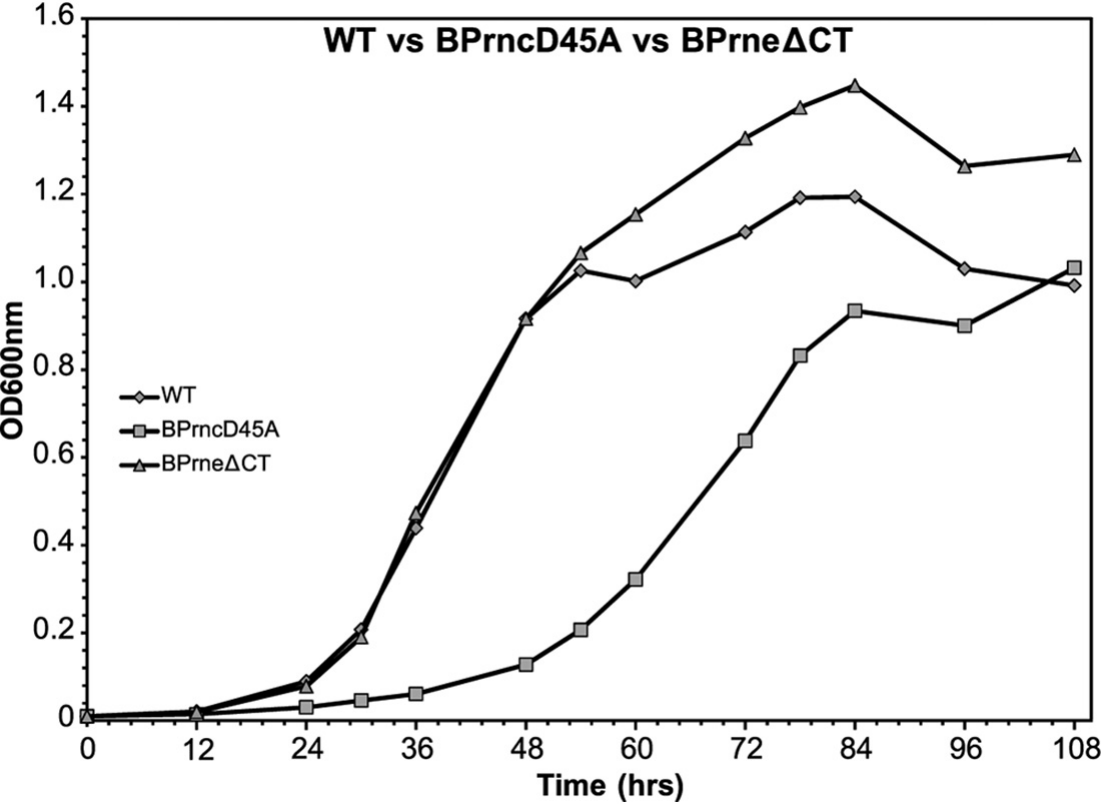
Comparison of the growth kinetics of the wild type (♦), BPrncD45A (■), and BPrneΔCT (▴) strains. Growth rates were consistent over 4 independent experiments. A representative experiment is shown. BPrncD45A and BPrneΔCT refer to the RNase III and RNase E mutant, respectively.

When grown on Bordet-Gengou (BG) agar, the B. pertussis RNase III mutant (BPrncD45A) produced hemolytic colonies which appeared to be smaller than wild type. This growth defect was further observed when the strain was grown in complete Stainer-Scholte medium (SS-medium; Fig. 1), with the RNase III mutant strain showing an extended lag phase yet reaching comparable optical densities to wild type over the course of the growth curve. This growth defect was reversed through complementation with the wild-type copy of the gene constitutively expressed from a low-copy-number plasmid (Fig. S2A).

### Generation and growth characteristics of the RNase E mutant, BPrneΔCT.

The turnover of mRNAs targeted by sRNAs is efficiently catalyzed by the endonuclease RNase E. Through interactions with the RNase E C-terminal half, the RNA chaperone Hfq bound to an sRNA is proposed to assist in directing the endonuclease to target transcripts for degradation (39). RNase E is an essential RNase required for the maturation of ribosomal RNAs and tRNA precursors (8). Despite the essentiality of RNase E, investigators working with E. coli and Salmonella have established viable RNase E mutations, allowing studies of the enzyme *in vivo* (40, 41). Indeed, mutations resulting in the truncation of the RNase E C-terminal domain have been shown to reduce RNase E function without negatively impacting the enzyme’s role in rRNA maturation (42). Importantly, the RNase E C-terminal half is required for mRNA targeting mediated by Hfq and small RNAs (15).

To understand more about the role RNase E plays in transcript stability and turnover in B. pertussis, 465 amino acids were deleted from the C-terminal half of RNase E to generate the strain BPrneΔCT (Fig. S1B). This mutation was designed to mimic the *rne*-105 mutation in E. coli (42). When grown in SS-medium, the mutant strain grew to an optical density greater than that of the wild-type strain and aggregated less in liquid medium (Fig. 1). When grown on BG agar, the colonies of the RNase E mutant were similar in size to those of the wild-type strain from which it was derived; however, the RNase E strain was nonhemolytic. This phenotype was reversed when complemented with the full-length protein (Fig. S2B and C).

10.1128/mSphere.00650-21.1 FIG S1 Schematics showing the mutations made in the B. pertussis *rnc* and *rne* genes. (A) Alignment of predicted amino acid sequence of the B. pertussis *rnc* gene with *A. aeolicus*, E. coli, and S. coelicolor *rnc*. The mutated residue at D45 is indicated by a black arrow. Schematic of the mutation introduced to the *rnc* gene is shown below the alignment. (B) Alignment of the predicted amino acid sequence of the B. pertussis *rne* gene with E. coli and *S. typhimurium rne.* Black arrow indicates the last amino acid within the open reading frame. This residue maps to the rne-105 mutation in E. coli, in which a frame shift mutation results in a premature stop (42). Schematic of the mutation introduced into the *rne* gene on the B. pertussis chromosome is shown below the alignment. Alignments for both endonucleases and homologues were run using Clustal Omega (Madeira F, Park YM, Lee J, Buso N, Gur T, Madhusoodanan N, Basutkar P, Tivey ARN, Potter SC, Finn RD, Lopez R. 2019. Nucleic Acids Res 47:W636-W641), and visualized using ESPript3 (Robert X, Gouet P. 2014. Nucleic Acids Res 42:W320-W324). Download FIG S1, TIF file, 1.8 MB. Copyright © 2021 Ifill et al. 2021 Ifill et al. https://creativecommons.org/licenses/by/4.0/ This content is distributed under the terms of the Creative Commons Attribution 4.0 International license.

10.1128/mSphere.00650-21.2 FIG S2 Complementation of endonuclease mutants. (A) Growth kinetics of *rncD45A* complemented with B. pertussis *rnc* in pBBR2pcpn (**×**) compared to BP338 wild type (♦) and BPrncD45A (■). (B) Hemolytic activity of BP338 wild type (top left), BPrneΔCT (bottom left), BPrneΔCT complemented with empty vector (bottom right), and BPrneΔCT complemented with B. pertussis *rne* in pBBR2pcpn (top right). (C) Immunoblots of BPrneΔCT and BPrneΔCT + pBBR2pcpn-*rne*, showing restoration of adenylate cyclase (CyaA) expression. Cultures grown on BG plates were resuspended in 1 ml SS salts and adjusted to an OD_600_ of 1.0. Expression of CyaA was then detected using the immunoblot procedure described in the Materials and Methods. (D) RT-qPCR of complemented endonuclease mutants showing restoration of transcript stability in *brkA* and *tcfA*. RNA was extracted from wild type and endonuclease mutants and complemented strains grown to mid log phase (OD_600_ .6 to 0.8). The bar graph shows the mean of the relative expression of *brkA* and *tcfA* compared to wild type plotted on a log_2_ *y* axis. Error bars show standard deviation of 3 replicates. A representative of 2 experiments is shown. (E) Immunoblots of BPrncD45A and BPrneΔCT complemented with pBBR2pcpn carrying wild-type copies of *rnc* or *rne*, respectively. Samples were taken from cultures grown on plates. Expression of BrkA and TcfA was analyzed using antisera raised against each protein. Download FIG S2, TIF file, 3.0 MB. Copyright © 2021 Ifill et al. 2021 Ifill et al. https://creativecommons.org/licenses/by/4.0/ This content is distributed under the terms of the Creative Commons Attribution 4.0 International license.

### Impact of mutations in RNase III and RNase E on the B. pertussis transcriptome.

The mutations generated in the B. pertussis *rnc* and *rne* genes were designed to alter RNA turnover in the cell. Overall, this would impact RNA metabolism as well as many systems associated with posttranscriptional regulatory circuits. To determine the changes to the regulation of the B. pertussis transcriptome, the wild-type and mutant strains were grown to mid-log phase (optical density at 600 nm [OD_600_] = 0.6 to 0.7) and RNA was extracted for transcriptomic analysis by RNA-Seq. Sequencing reads were aligned to the Tohama I genome (NCBI:txid257313) (33) and gene expression changes were compared to wild type using DESeq2 (43). Differentially expressed genes were defined as having a fold change of ≥1.5 compared to wild type and an adjusted *P* value ≤ 0.05 across five biological replicates.

**FIG 2:**
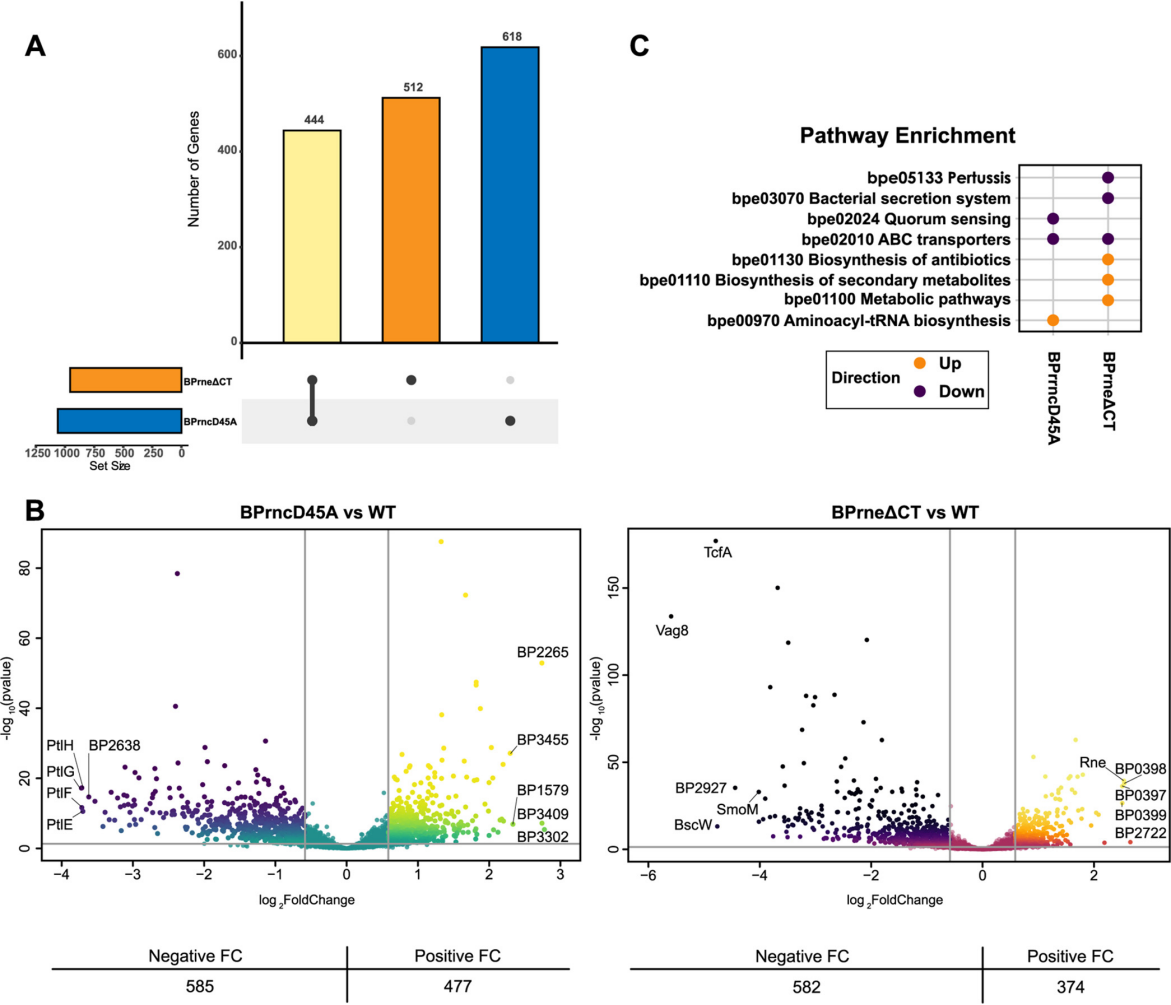
Differential gene expression in the RNase III (BPrncD45A) and RNase E (BPrneΔCT) mutants compared to wild type as determined by RNA-Seq. (A) UpSet plot showing the total number of genes differentially expressed in each mutant strain compared to wild type. The total number of differentially expressed genes in BPrncD45A (1,062) and BPrneΔCT (956) are indicated on the scale at the bottom left of the plot. Genes unique to each set are indicated by a filled circle and genes shared in differential expression are indicated by filled circles connected by a bar. The number of differentially expressed genes in each subset is indicated above each histogram. (B) Volcano plots illustrating the range of differentially expressed genes from transcriptomic analysis of BPrncD45A mutant (left) and the BPrneΔCT mutant (right). Differential expression cutoffs showing fold change of ≤ or ≥ 1.5× compared to wild type and a *P* value ≤ 0.05 are indicated by the gray lines. All genes identified in the RNA-Seq analysis are plotted. The total number of genes showing a significant positive or negative fold change (FC) relative to wild type in each mutant strain is indicated below the plots. The 5 most differentially expressed genes (positive and negative FC) for each data set are labeled in the plots. (C) GAGE analysis determining KEGG pathway enrichment in the endonuclease mutants. Enriched pathways are indicated by a filled circle indicating downregulation (purple) or upregulation (orange) of the associated KEGG pathway.

In the RNase III mutant, there were 1,062 genes differentially expressed when compared to the wild type (Fig. 2A). As shown in volcano plots (Fig. 2B) mapping the spread of differentially expressed genes, the expression of 585 of these genes was negatively affected by the loss of RNase III activity, while 477 were upregulated. Compared to wild type, *ptlEFGH* were among the most downregulated genes in this data set, while the type three secretion system (T3SS) chaperone protein bp2265 was among the most upregulated (Table S1). Specific pathways, identified using Generally Applicable Gene-set Enrichment (GAGE) (44), were shown to be dysregulated in the RNase III mutant (Fig. 2C). This included pathways associated with quorum sensing (bpe02024), ABC transporters (bpe02010), and tRNA biosynthesis (bpe00970).

The RNase E mutant showed 956 genes differentially expressed when compared to wild type (Fig. 2A). Like the RNase III mutant, more than 580 genes were downregulated by the mutation to RNase E (Fig. 2B). Comparing the two data sets, 444 genes were commonly differentially expressed and, of these, 131 genes (Table S1) showed opposing patterns in fold change in the two mutant backgrounds. Here, the autotransporters *vag8* and *tcfA* were the most downregulated genes, showing a >25-fold reduction when compared to wild type (Table S1). Interestingly, the *rne* gene itself was among the most upregulated genes, suggesting a potential impact on RNase E autoregulation (8). The RNase E mutant also showed enrichment in pathways associated with B. pertussis pathogenesis (bpe05133), bacterial secretion (bpe03070), and metabolic pathways (bpe01100) (Fig. 2C).

10.1128/mSphere.00650-21.3 TABLE S1 Differentially expressed genes found in BPrncD45A and BPrneΔCT compared to WT. Download Table S1, XLSX file, 0.3 MB. Copyright © 2021 Ifill et al. 2021 Ifill et al. https://creativecommons.org/licenses/by/4.0/ This content is distributed under the terms of the Creative Commons Attribution 4.0 International license.

RNase E is involved in the processing of sRNAs in various bacterial pathogens, yet RNase III can also play a role in sRNA turnover due to RNA duplex formation with the target mRNA (45). Several putative targeted sRNAs have been previously identified by *in silico* analysis of the B. pertussis genome (46). Using the predicted genome coordinates of these 17 sRNAs, we examined whether the endonuclease mutations altered the expression of this subset of putative regulatory RNAs in B. pertussis. Even though sRNAs are typically processed by RNase E, it was surprising that only two of the predicted sRNAs (*bprK* and *bprM’*) were differentially expressed in the RNase E mutant, although four sRNAs (*bprB*, *bprJ*, *bprM*, and *bprM’*) were found to be differentially expressed in the RNase III mutant. One of these sRNA species, *bprM’*, was differentially expressed in both mutants, but it was upregulated in the RNase III mutant and downregulated in the RNase E mutant (Table S1).

Taken together, the endonuclease mutations each affected approximately one quarter of the B. pertussis transcriptome. Even though each mutation ultimately impairs endonuclease function, only approximately half of the differentially expressed transcripts appeared to become less abundant through the loss of full RNase III or RNase E activity. This data suggests that these endonucleases may contribute to stabilizing many mRNAs within the B. pertussis transcriptome. Another outstanding observation was the substantial variation, and moderate overlap between the impacts of these two endonucleases, implying novelty with a degree of redundancy.

### Validation of RNA-Seq data set.

**FIG 3:**
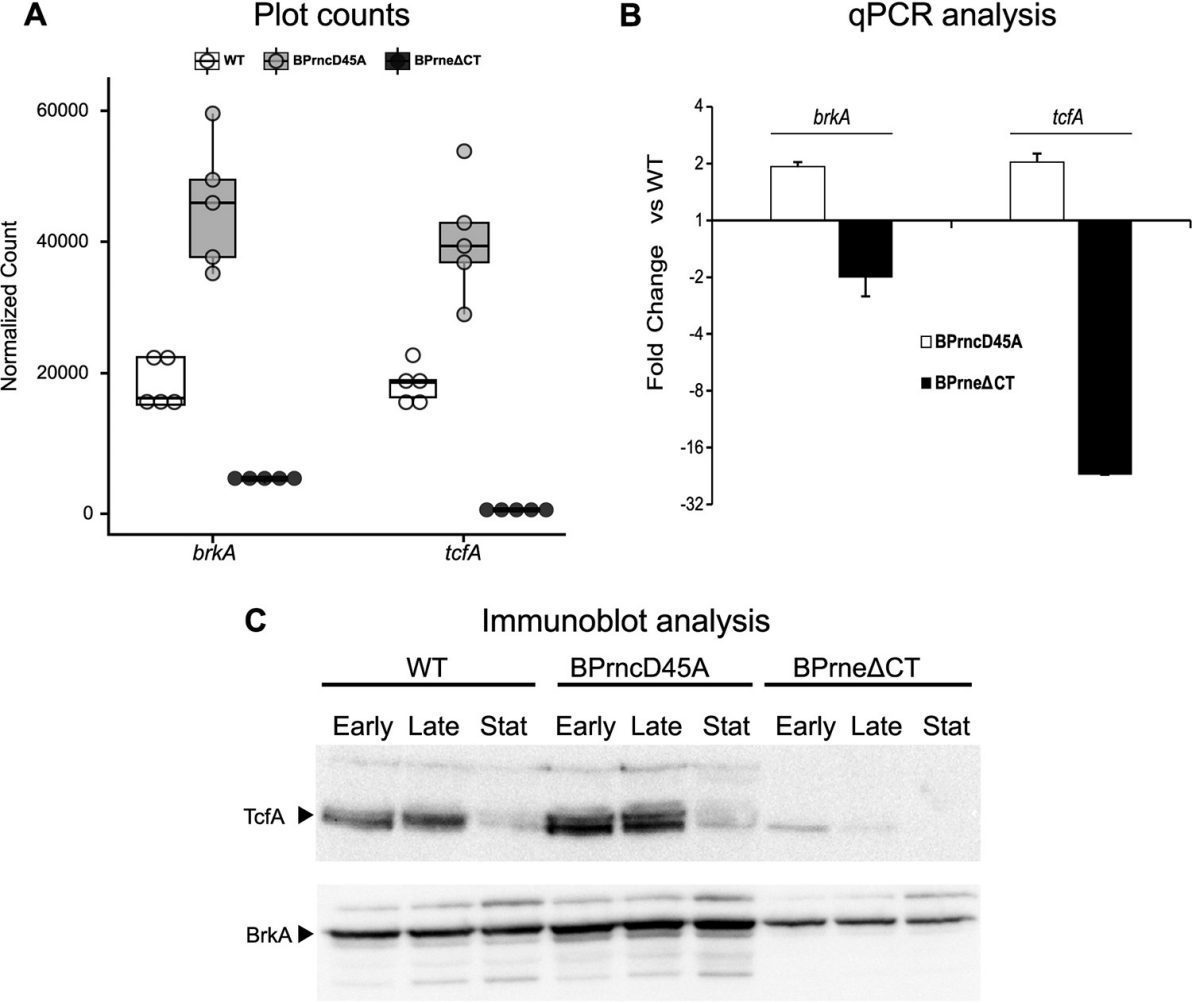
Validation of the RNA-Seq data sets using *brkA* and *tcfA*. (A) Normalized counts of *brkA* and *tcfA* transcripts as reported from transcriptome analysis of wild-type B. pertussis and BPrncD45A and BPrneΔCT. Box plots indicate the number of reads for each gene across 5 biological replicates. (B) Validation of the RNA-Seq data by qPCR. RNA was extracted from wild type and endonuclease mutants in late log phase. The bar graph shows the mean of the relative expression of *brkA* and *tcfA* compared to wild type plotted on a log_2_ *y* axis. Error bars show the standard deviation across 3 replicates. A representative of three experiments is shown. (C) Validation of RNA-Seq data by immunoblotting. Samples were taken at growth phases indicated above each lane (Early, early log phase; Late, late log phase; Stat, stationary phase). Expression of BrkA and TcfA was assessed using antisera raised against each protein. Reduction and absence of TcfA in stationary phase for the three strains is due to the protein being released from the cell surface (77).

To verify the gene fold changes reported in the RNA-Seq data set, we experimentally characterized the differential expression of two genes in the mutant backgrounds. Specifically, we examined the expression changes of the autotransporters BrkA and TcfA, virulence factors that were also shown to be differentially expressed in the Δ*hfq* mutant (28). Fig. 3A demonstrates the normalized counts of both *brkA* and *tcfA* and shows a near 2-fold increase in transcript abundance in the RNase III mutant. A stronger phenotype was observed in the RNase E mutant, where there was a 4-fold reduced abundance of *brkA* transcripts and, strikingly, a normalized count for *tcfA* approaching zero, which translated to a 28-fold reduction in transcript abundance compared to wild type (Table S1). To verify these data at the RNA and protein level, fresh cultures were inoculated into SS-medium and samples for quantitative PCR (qPCR) were taken at late log phase for RNA analysis. For protein expression, samples for immunoblots were taken across a time course at early log phase, late log phase, and stationary phase. The results from qPCR showed an approximately 2-fold increase in *brkA* and *tcfA* transcripts in the RNase III mutant compared to wild type. In the RNase E mutant, qPCR showed a 2-fold and 20-fold reduction in *brkA* and *tcfA*, respectively (Fig. 3B). Differences in protein abundance were estimated by analyzing the density of bands corresponding to expression of BrkA and TcfA in immunoblots. Using ImageJ (47) for quantification, the RNase III mutant showed an approximate 1.5-fold increase in BrkA and TcfA levels when compared to wild type. In the RNase E mutant, protein expression of BrkA was reduced by approximately 2-fold, whereas TcfA protein expression was almost nondetectable across the time course (Fig. 3C). These data thus corroborated the RNA-Seq analysis by showing gene and protein expression patterns that mirrored what was seen in the transcriptome analysis of the two mutants. Notably, phenotypes for each endonuclease mutant at both the RNA (qPCR) and protein (immunoblot) levels were returned to wild-type levels upon complementation with the wild-type copy of the appropriate gene (Fig. S2D and E).

### Mutations in RNase III and RNase E drove virulence factor stability in opposite directions.

The BvgAS two-component regulatory system sits at the center of virulence gene expression in *Bordetella*. In the Bvg+ mode, more than 500 genes are regulated by phosphorylated BvgA (Table 1) (48), including several genes associated with virulence. Since both RNase III and RNase E are implicated in bacterial virulence factor production and pathogenesis (49), they might influence or be influenced by the BvgAS regulon. Comparison between the RNA-Seq data set collected here and the recent transcriptomic analysis of the BvgAS regulon (21) showed that approximately 50% of the genes in the BvgAS regulon were differentially expressed in the RNase III and RNase E mutant strains (Table 1, Table S2 and S3).

**TABLE 1:** Number of genes found to be differentially expressed in BPrncD45A and BPrneΔCT which are also found to be differentially expressed in transcriptomic analyses of the *BvgAS* regulon (21) and the *hfq* regulon (28)^*a*^

Data set	No. of genes differentially regulated		
	BvgAS or Hfq regulon	BPrncD45A	BPrneΔCT
BvgAS regulon			
BvgAS upregulated	237	111	144
BvgAS downregulated	322	155	145
BvgAS-associated metabolism genes	119	69	64
Well characterized VAGS	71	36	66
Hfq regulon			
Hfq upregulated	156	96	93
Hfq downregulated	212	86	88

a Data sets from these studies were chosen due to similarities in growth conditions of wild-type and mutant strains.

10.1128/mSphere.00650-21.4 TABLE S2 Genes upregulated by BvgAS found to be differentially expressed in the endonuclease mutants. Download Table S2, XLSX file, 0.02 MB. Copyright © 2021 Ifill et al. 2021 Ifill et al. https://creativecommons.org/licenses/by/4.0/ This content is distributed under the terms of the Creative Commons Attribution 4.0 International license.

10.1128/mSphere.00650-21.5 TABLE S3 Genes downregulated by BvgAS found to be differentially expressed in the endonuclease mutants. Download Table S3, XLSX file, 0.02 MB. Copyright © 2021 Ifill et al. 2021 Ifill et al. https://creativecommons.org/licenses/by/4.0/ This content is distributed under the terms of the Creative Commons Attribution 4.0 International license.

**FIG 4:**
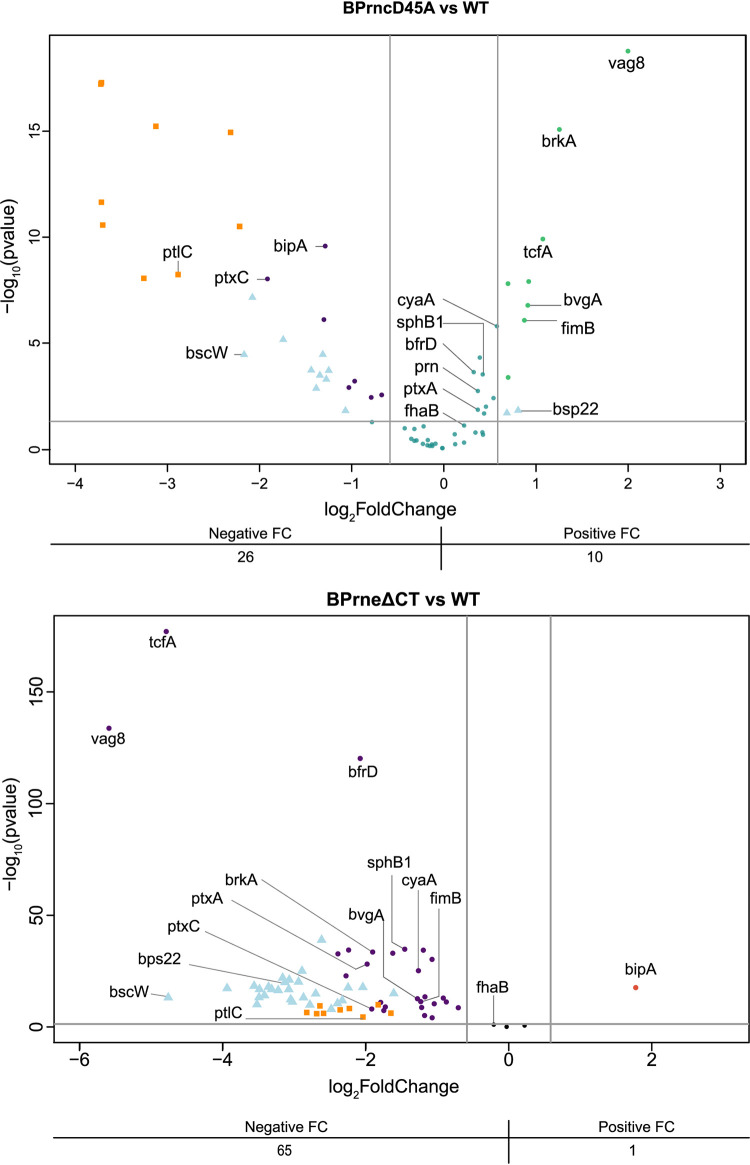
RNase III and RNase E have opposing activities on virulence factor transcripts. Depiction of volcano plots filtered to show differential expression of well-characterized virulence factors (21) in BPrncD45A (top) and BPrneΔCT (bottom). In both plots, significantly differentially expressed genes of the type three secretion locus and pertussis toxin liberation operon are indicated by triangles (blue) and squares (orange), respectively. Plots map 69 of the 71 genes in the subset; BP1880 and BP2260 were not detected from our RNA-Seq analysis. FC refers to fold change.

B. pertussis expresses several virulence factors in the Bvg+ mode, with many characterized through *in vitro* and *in vivo* infection models (48). As part of the BvgAS regulon, these well-characterized virulence factors number approximately 70 genes and comprise those encoding toxins, secretion systems, and colonization factors. To determine whether the mutations in RNase III and RNase E impacted the stability of these virulence gene transcripts, the fold changes of these transcripts were identified within our RNA-Seq data set. Fig. 4 and Table S4 detail the fold changes of the 71 well-characterized virulence genes in the two endonuclease mutants. Interestingly, 66 of these virulence factor transcripts were found to be less abundant in the RNase E mutant, including the *cya* operon, which is likely responsible for the nonhemolytic phenotype observed with this mutant. Autotransporters *tcfA* and *vag8*, and the putative type 3 secretion chaperone *bscW*, were more than 25-fold downregulated when compared to wild type. In the RNase III mutant, 36 of these 71 virulence factors were significantly differentially expressed. Of these virulence factors, 10 were found to be more abundant when compared to wild type and shown to be less abundant in the RNase E mutant; however, it should be noted that the *ptl* locus was downregulated in both mutant backgrounds. The BvgAS system can also produce a third gene expression profile intermediary of Bvg+ and Bvg− known as the Bvg intermediate mode (Bvg^i^) (23). The marker protein of the Bvg^i^ phase, BipA, showed a similar pattern in transcript abundance wherein its transcript abundance was reduced in the RNase III mutant yet increased in RNase E mutant. Finally, although the Tohama-1 strain of B. pertussis does not have a functional T3SS when grown *in vitro* (i.e., in the absence of host cells) (28, 50), transcripts associated with the T3SS locus were less abundant in both mutants, with the tip complex protein Bsp22 being more abundant in the RNase III mutant.

Taken together, these data identify an interesting relationship in which RNase III and RNase E have opposing activities on select virulence factor transcripts. This association suggests these endonucleases may be involved in modulating transcript stability during pathogenesis as a potential means of fine-tuning virulence factor expression within the host.

### RNase III and RNase E processed transcripts associated with metabolism in the Bvg+ mode.

During the Bvg+ mode, phosphorylated BvgA alters the expression of many genes associated with metabolic and catabolic pathways. This suggests that B. pertussis coordinates substantial changes to cell homeostasis between Bvg+ and Bvg− modes. Since both RNase E and RNase III are associated with controlling metabolic gene expression in E. coli (9), we sought to identify differentially expressed metabolism genes associated with the Bvg+ mode (21).

**FIG 5:**
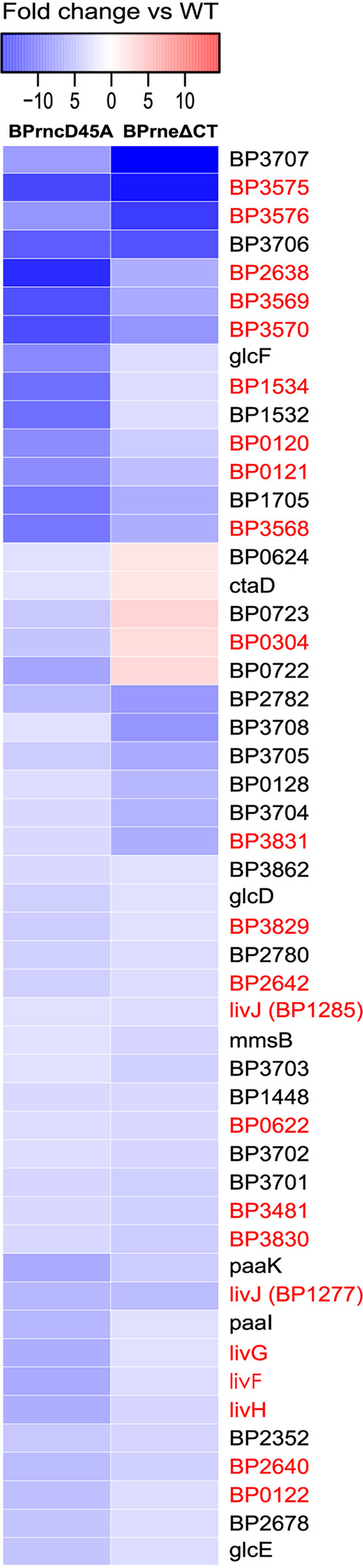
Heatmap showing the average fold changes of the 50 genes associated with B. pertussis metabolism in the Bvg+ mode differentially expressed in both the BPrncD45A mutant (left) and the BPrneΔCT mutant (right). Gene names shown in red indicate ABC transporters associated with KEGG pathway bpe02010.

This comparison showed that 69/119 of the Bvg-associated metabolism genes were also affected by the mutation to RNase III. Of this subset, all genes associated with the RNase III mutation were less abundant (Fig. 5). We performed the same analysis on the RNase E mutant and showed that 64/119 of the Bvg-associated metabolism genes were differentially regulated. Of these 64 differentially expressed genes, 50 were shared between the RNase E and RNase III mutants and the transcripts associated with many members of this gene subset were less abundant compared to wild type (Fig. 5). Further analysis showed that 23 of these 50 genes were ABC transporters associated with amino acid uptake (Fig. 5, Table S5) found in GAGE pathway bpe02010 (Fig. 2C).

10.1128/mSphere.00650-21.6 TABLE S4 Well-characterized virulence factors found to be differentially expressed in the endonuclease mutants. Download Table S4, XLSX file, 0.02 MB. Copyright © 2021 Ifill et al. 2021 Ifill et al. https://creativecommons.org/licenses/by/4.0/ This content is distributed under the terms of the Creative Commons Attribution 4.0 International license.

10.1128/mSphere.00650-21.7 TABLE S5 Metabolism-associated genes regulated by BvgAS found to be differentially expressed in the endonuclease mutants. Download Table S5, XLSX file, 0.02 MB. Copyright © 2021 Ifill et al. 2021 Ifill et al. https://creativecommons.org/licenses/by/4.0/ This content is distributed under the terms of the Creative Commons Attribution 4.0 International license.

Taken together, these data suggest that both RNase III and RNase E play a role in the maintenance of many metabolism transcripts in the Bvg+ mode. Since many of the genes involved appeared to be associated with resource uptake, the data suggests that these endonucleases may somehow be involved in coordinating the transition in cell homeostasis between Bvg+ and Bvg− modes.

### Comparative analysis of B. pertussis transcriptomic data provided insights into regulatory RNA networks.

Previous work in B. pertussis has identified several regulatory RNAs, yet not much is known about the target transcripts on which they act. As endonucleases are often involved in the processing of the target mRNA coupled with regulatory RNAs, we compared the findings from the transcriptomic analysis of the endonuclease mutants here to previously published studies regarding the B. pertussis primary transcriptome (30) and the *hfq* regulon (28) to identify putative gene loci which might be regulated by either asRNAs or sRNAs.

As recently detailed in the report of the B. pertussis primary transcriptome (30), a majority of the asRNA transcripts are produced from internal promoters found within the large number of insertion (IS) elements present within the B. pertussis genome. Transcription from promoters present within the insertion elements has been shown to run into the adjacent genes. In some cases, these are found in antisense to the adjacent gene and can influence transcript stability (51). Since RNase III is involved in cleaving double-stranded RNA, we investigated whether RNase III was involved in the resolution or degradation of transcripts at IS antisense junctions.

The B. pertussis primary transcriptome identified 192 genes adjacent to IS elements and influenced by an antisense transcript (30). Of these, 39 genes (20%) were found to be differentially expressed in the RNase III mutant (Table S6). Genes such as *fim2*, *metX*, and *recR,* as well as other genes in this subset, were less abundant when compared to wild type, suggesting that endonuclease RNase III may be required to stabilize the interacting transcripts produced from these loci.

We also compared our findings to the regulon controlled by the RNA chaperone Hfq, since Hfq plays a substantial role in mediating the interactions, stability, and processing of sRNAs with their mRNA targets (27, 28). This analysis established that approximately 50% of the genes in the *hfq* regulon were differentially expressed in both endonuclease mutants (Table 1, Table S7). This overlap of genes included many ABC transporters and virulence factors, including the autotransporters *brkA*, *tcfA*, and *vag8*. Moreover, many genes required for the type III secretion system were downregulated in all three mutant backgrounds. Given this, we looked for additional operons that may be influenced in a similar way. This analysis identified 19 unique operons (Table S8), including several ABC transporters, a quinol oxidase biosynthesis cluster, and the *ptl* operon required for pertussis toxin secretion (52).

10.1128/mSphere.00650-21.8 TABLE S6 Identification of differentially expressed genes adjacent to IS elements influenced by an antisense transcript. Download Table S6, XLSX file, 0.02 MB. Copyright © 2021 Ifill et al. 2021 Ifill et al. https://creativecommons.org/licenses/by/4.0/ This content is distributed under the terms of the Creative Commons Attribution 4.0 International license.

10.1128/mSphere.00650-21.9 TABLE S7 Genes differentially expressed in the B. pertussis Δhfq mutant were also differentially expressed in the BPrncD45A and BPrneΔCT mutants. Download Table S7, XLSX file, 0.03 MB. Copyright © 2021 Ifill et al. 2021 Ifill et al. https://creativecommons.org/licenses/by/4.0/ This content is distributed under the terms of the Creative Commons Attribution 4.0 International license.

Thus, using the known relationship between RNase E and Hfq and the specific activity for double-stranded RNA by RNase III, our work identified a number of gene loci that were previously not known to be regulated at the posttranscriptional level.

## DISCUSSION

Regulatory RNAs provide bacterial pathogens with a mechanism to rapidly modify gene expression profiles while under stress. Often processed by one of several ribonucleases, cleavage of the target mRNA can substantially alter the stability of the transcript and/or modulate rates of translation. RNase III and RNase E are endonucleases involved in processing of *cis*-encoded asRNA and *trans*-encoded sRNAs, respectively. Here, mutants of both RNase III and RNase E were generated by allelic exchange and the impact of these mutations on the B. pertussis transcriptome was assessed by RNA-Seq.

The mutations to RNase III and RNase E resulted in the differential expression of approximately 1,000 genes, representative of about 25% of the B. pertussis genome. Figure 2B shows that many of the transcripts found to be differentially expressed in the two mutants were less abundant when compared to wild type. With the impaired activity in both endonuclease mutant backgrounds, we expected to observe increases in transcript abundance. However, it was surprising to see more than half of the differentially expressed transcripts were reduced in abundance in the mutant strains. In the case of the RNase III mutation, this reduction in transcript abundance might have been, in part, a consequence of the slow growth phenotype associated with the mutation (53). For example, much of the lipid A biosynthesis pathway genes (*lpxABDHK*) were approximately 2-fold reduced when compared to wild type (Table S1), corresponding to the approximately 2-fold reduction in the doubling rate of the mutant cells. In contrast, there was no growth defect associated with the C-terminal truncation of RNase E, and yet a similar proportion of transcripts were reduced in BPrneΔCT, the RNase E mutant strain. Interestingly, the Lgm locus (locus tags BP0397 to BP0399), which is involved in the modification of lipid A (54), was increased almost 6-fold in the RNase E mutant (Fig. 2B). Changes in transcript abundance may be associated with several mechanisms involved in RNA half-life, such as changes in rates of translation and a loss of cleavage events mediated by these two endonucleases, resulting in alternate RNA secondary structures (55). Differential processing of regulatory RNAs may also contribute to the stability of these transcripts (56).

RNase E is associated with the turnover of sRNAs in bacteria through the C-terminal domain coordinating interactions with the RNA chaperone Hfq (57). RNase III is also involved in regulatory RNA circuits by cleaving RNA-RNA duplexes, such as those formed from asRNA binding to its mRNA target (4, 58). Taking our RNA-Seq data set, we compared the data to other published studies to establish associations between the endonuclease mutants and known sRNAs and asRNAs. Using this approach, we showed that 5 previously identified sRNAs (46) were differentially expressed in our data set. Of these, 4 of the sRNAs were unique to the RNase III mutant. Even though RNase III is typically associated with cleaving asRNAs, this endonuclease also processes *trans*-encoded regulatory RNAs (11, 12, 59, 60). Furthermore, as the RNase III mutation influenced many metabolic processes, this might have resulted in several stress responses contributing to the production of sRNAs enriched under these conditions (2, 5). It is interesting that RNase III also played a role in regulating many genes found to be in an antisense orientation to an adjacent insertion element. Many of these genes were downregulated in the mutant strain, suggesting that the full catalytic function of RNase III is potentially required for the activation of many genes near insertion elements.

One of the most striking phenotypes of the RNase E mutant was the loss of hemolysis (Fig. S2B) accompanied by marked reduction in expression of CyaA (Fig. S2C). Hemolysis in Bordetella pertussis is due to the activity of adenylate cyclase toxin, CyaA, which is produced and exported by the genes found in the *cya* operon (61). The RNA-Seq analysis here showed that in the RNase E mutant, the *cya* operon was approximately 2-fold reduced compared to wild type. This would suggest a decrease in hemolysis, although in fact a complete absence was observed. Interestingly, diminished adenylate cyclase activity was also observed in the Δ*hfq* strain (27). Of note, qPCR analysis of *cyaA* in this *hfq* mutant showed that transcript abundance was similar to the wild-type strain, implying that the cyaA mRNA requires posttranscriptional modification for optimal translation. Taken together, this suggests that RNase E is likely involved in processing of the *cyaA* transcript in an Hfq-dependent manner. Hfq is known to catalyze the interactions with a small RNA (15, 16); however, the associated regulatory RNA involved with modulating *cya*A translation is yet to be identified. Additionally, the reduction in the *cya* operon transcript abundance in the RNase E mutant may also suggest that this endonuclease is involved in stabilizing the mRNA of the entire locus. Overall, this provides several layers of complexity around the regulation of adenylate cyclase toxin expression in B. pertussis, although the importance of this regulation in *Bordetella* pathogenesis is yet to be determined.

To identify loci potentially regulated by regulatory RNAs, we compared the transcriptomes of each endonuclease mutant to the *hfq* regulon (28). This revealed a substantial overlap with genes that were also differentially expressed in the mutant *hfq* background. From this, it was observed that much of the T3SS was downregulated in both the RNase III and RNase E mutant strains, as well as in the Δ*hfq* strain, further implying a role for regulatory RNAs in the function of the secretion system. In many bacteria, the control of the T3SS tends to be complex and both regulatory RNAs and endonucleases have been implicated in this process (62). In *Yersinia*, both RNase E (63) and Hfq (64, 65) were shown to play a role in the function of the secretion system. Among the *Bordetella* spp., regulation of T3SS consists of the extracytoplasmic sigma factor BtrS, a cognate anti-sigma factor BtrA, and partner-switching modules BtrVW (66). In both the endonuclease mutants here described, these regulatory proteins were differentially expressed compared to wild type under these conditions. However, there were no significant changes in transcript abundance of BtrASVW in the *hfq* background (28). These data add an additional layer of complexity to the regulation of the T3SS, potentially involving factors outside the T3SS locus. Further investigation would be required to determine a mechanism by which this may work.

Alongside the T3SS, more than 90% of widely studied virulence factors involved in B. pertussis pathogenesis (21) were decreased in abundance in the RNase E mutant, with more than half of these well-characterized virulence factors being more than 4-fold reduced, and some more than 20-fold reduced in abundance. This was quite a striking finding, since similar mutations in E. coli are often associated with a more subtle impact on transcript stability (42). Surprisingly, many of these virulence factors were upregulated in the RNase III mutant. Of the 444 genes that shared differential expression in both mutants, 131 of these genes had opposing fold changes in the two mutant backgrounds. The opposite effects on transcript stability produced by these two endonucleases have been observed previously; however, the reason for this remains unclear (56). Gene transcripts showing opposite stabilities in the mutant backgrounds include the response regulator b*vgA* and autotransporters *vag8*, *tcfA*, and *brkA*. It is intriguing that the mutations in RNase III and RNase E exerted such a drastic impact on the transcripts for *tcfA* and *vag8*, since both these autotransporters were also strongly downregulated in the *Δhfq* strain of B. pertussis (27, 28). Taken together, these data suggest that the abundance of these autotransporter transcripts could be determined by regulatory RNAs. Complex regulatory interactions involving RNase III, RNase E, and Hfq have been characterized previously (56, 60, 67). For example, the inactivation of the *sodB* transcript in E. coli under iron-limited conditions requires RNase III, RNase E, and the sRNA *RyhB* in complex with Hfq (59). However, the regulatory RNA influencing expression of *vag8* and *tcfA* in B. pertussis, and the mechanism by which it interacts with these genes, are yet to be elucidated.

Currently, there are few well-defined mechanisms by which regulatory RNAs work in B. pertussis. The work here highlights additional regulation of metabolism and key virulence factors involved in B. pertussis pathogenesis. Furthermore, by comparing our data to the currently available data on the B. pertussis transcriptome, we have highlighted gene loci potentially influenced by regulatory RNAs, including the Lgm locus (54), which we are currently investigating. Even though there are other ribonucleases present in B. pertussis, the mutant strains generated here provide details on the posttranscriptional landscape of B. pertussis while also contributing another layer of understanding of the complex regulatory circuits responsible for *Bordetella* pathogenesis.

## MATERIALS AND METHODS

### Bacterial strains, plasmids, and growth conditions.

All strains used in this study are detailed in Table 2. Lysogeny broth (LB; tryptone 10 g/liter, yeast extract 5 g/liter, NaCl 10 g/liter) or LB plus agar was used for the growth of Escherichia coli strains. *Bordetella* strains were grown either on Bordet-Gengou (BG; BD Difco) agar plates supplemented with 15% defibrinated sheep’s blood (Dalynn Biologicals) or in complete Stainer-Scholte (SS) medium (68) supplemented with 0.15% bovine serum albumin (BSA; Sigma) (69). Growth of bacterial strains was performed at 37°C. As needed, media were supplemented with nalidixic acid (30 μg/ml), gentamicin (15 μg/ml), and/or diaminopimelic acid (DAP) (250 μg/ml).

**TABLE 2:** Strain, plasmid, and primer list

Strain, plasmid, or primer name	Description or sequence	Reference/source/notes
E. coli strains		
DH5α	Molecular cloning strain	
RHO3	Conjugation strain; Km^s^ Δasd ΔaphA, DAP auxotroph	78
B. pertussis strains		
BP338 (WT)	Wild-type B. pertussis Tohama-1 strain; Nal^r^	79
BP338rncD45A	B. pertussis containing catalytically inactive RNase III allele, *rnc*D45A; Nal^r^	This study
BP338rneΔCT	B. pertussis containing truncation of the C-terminal domain of RNase E, *rne*^1-578^; Nal^r^	This study
Plasmids		
pSS4894	Suicide vector containing I-SceI restriction enzyme and cognate restriction site, used for allelic exchange; Gm^r^	38
pGI-rncD45A	pSS4894 with up- and downstream fragments required for mutagenesis of rnc gene in B. pertussis	This study
pGI-rneΔCT	pSS4894 with up- and downstream fragments required for in frame deletion of rne C-terminus in B. pertussis	This study
pBBR2-pcpn	Broad host range, low copy no. plasmid, consists of cpn10 heatshock promoter adjacent to multiple cloning site.	54
pBBR2-rnc	pBBR2-pcpn with wild type B. pertussis rnc.	This study
pBBR2-rne	pBBR2-pcpn with wild type B. pertussis rne.	This study
Primers		
rncD45A 1_fw	aaaaaaACGCGTGTACATCAACGGAAAATTGGTGCC	Used in construction of pGI-rncD45A
rncD45A 1_rev	aaaaaaGCTAGCGCCGAGAAACTCCAGCCGCTCGTT	
rncD45A 2_fw	aaaaaaGCTAGCGTGCTGAACTTCGTCGTCGCGGCG	
rncD45A 2_rev	aaaaaaGGATCCGGCGGCGCGGCGGCTGGCGCCCGG	
rne-cterm_1 fw	aaaaaaGGATCCCGAAGGCTCGCACATCACCTGCCC	Used in construction of pGI-rneΔCT
rne-cterm 1_rev	aaaaaaGAATTCTTCGGTCTTGGCCGGCTCGGCGCT	
rne-cterm 2_fw	aaaaaaGAATTCCGCACAAAGCGGGTTGCGGCAGCG	
rne-cterm 2_rev	aaaaaaGTCGACCCAGGTGATGACCAACTCGCCGAC	
Rnc_fw	aaaaaGTCGACATTGACCGCTATGTCCCTTGCCACG	Used in construction of pBBR2-rnc
Rnc_rev	aaaaaaGGATCCTCATTTAACCTCTTGGGCCACTGC	
RNase E_fw	aaaaGAATTCATGAAGCGCATGCTGTTTAATGCGAC	Used in construction of pBBR2-rne
RNaseE_rev	aaaaaaGGATCCTCAGTGACGCGTTTCGACTTGCAC	
brkA-qpcr_fw	CGCAGGAGTTCAAAAGCACG	Used in qPCR of BrkA
brkA-qpcr_rev	GAATGTTGATCCGGTCGCC	
tcfA-qpcr_fw	GGTATGTGGACACTTTCTC	Used in qPCR of TcfA
tcfA-qpcr_rev	GCTTGAAATCCTCCAGAGACA	
RpoB-qpcr_fw	GCTGGGACCCGAGGAAAT	Used in qPCR of RpoB, housekeeping gene
RpoB-qpcr_rev	CGCCAATGTAGACGATGCC	

### Construction of endoribonuclease mutants in B. pertussis.

To generate the point mutation in the *rnc* gene on the B. pertussis chromosome, ∼500 bp upstream and downstream of the rncD45 codon were amplified using primers detailed in Table 2. Fragments were designed to be joined to each other using an NheI site. The position of the NheI site, when incorporated into the B. pertussis genome, would provide the desired D45A mutation, while not influencing the adjacent serine codon. This ∼1,000-bp fragment was then cloned into pSS4894 (38) between MluI sites and BamHI restriction sites to generate pGI-rncD45A. This construct was first transformed into E. coli RHO3 before conjugation into B. pertussis.

The truncation of the rne C-terminal half was generated by first amplifying ∼500-bp regions flanking the 1,398 bp of the *rne* C-terminal half that was to be removed. Flanking regions were ligated together at EcoRI sites and introduced to pSS4894 at SalI and BamHI restriction sites. This construct was transformed into RHO3 for conjugation.

Allelic exchange plasmids were delivered into B. pertussis by conjugation with minor alterations to the published procedure (38). Briefly, recipient strain BP338 was grown on BG agar in the presence of 50 mM MgSO_4_ and nalidixic acid for 3 days. These cells were then harvested from plates using a sterile swab and resuspended in 1.5 ml of SS broth supplemented with DAP. The optical density at 600 nm (OD_600_) of the resuspended BP338 was then measured. The donor RHO3 strains were grown overnight and OD_600_ was measured. BP338 and donor RHO3 strains were then mixed at a ratio of 2:1 and spotted onto SS-agar plates containing SS-supplements, BSA (0.15%), and 50 mM MgSO_4_. Conjugation mixtures were incubated for 6 h at 37°C before being swabbed and reswabbed onto fresh BG plates with nalidixic acid (30 μg/ml), gentamicin (100 μg/ml), and 50 mM MgSO_4_. After 4 days incubation at 37°C, single colonies were restreaked onto BG plates with nalidixic acid to allow activation of the BvgAS system and I-SceI expression driven by the pertussis toxin promoter. These plates were grown until distinct colonies could be observed. From this, single colonies were then screened by PCR for the presence of the mutant allele.

PCR-confirmed mutants were then grown for ∼72 h in complete SS-medium and genomic DNA was extracted using the DNeasy blood and tissue kit (Qiagen) as per the manufacturer’s instructions. Gene fragments, including upstream and downstream regions used for allelic exchange, were then amplified and the mutations confirmed by Sanger sequencing (Genewiz).

### RNA-Seq and bioinformatic analyses.

Wild-type and mutant cells were grown to an OD_600_ of 0.6 to 0.7 in SS broth and 2 ml of cells was harvested into 4 ml RNA Protect Bacteria (Qiagen). RNA was extracted from cell pellets using the RNAqueous RNA extraction kit (Ambion) following kit instructions, and DNA was then removed using the Turbo DNase kit. RNA concentrations were measured by nanodrop spectrophotometer and DNA removal was verified by PCR. Total RNA quality was assessed using a BioAnalyzer 2100, with each sample producing an RNA integrity number greater than nine.

rRNA depletion was completed using the RiboZero bacteria kit (Illumina). The KAPA Stranded Total RNA kit (KAPA Biosystems) was used for library construction (five replicates per genotype), and sequencing was done by the University of British Columbia’s Sequencing and Bioinformatics Consortium on an Illumina HiSeq2500. Single-end 100-bp reads were checked for quality using FastQC v0.11.7 (70) and aligned to the B. pertussis sp. strain Tohama I reference genome from NCBI (NCBI: txid257313 [33]) using the alignment program STAR v2.6.1a (71). Counts of aligned reads were generated using HTSeq’s count function v0.9.1 (72).

Raw library sizes for all samples had a minimum of 1,032,961, median of 4,355,770, and maximum of 8,888,916 reads after removing low count genes (i.e., fewer than 10 counts across the number of biological replicates). Differentially expressed genes between each of the mutants and wild-type bacteria were determined using the Wald statistical test implemented in the Bioconductor package DESeq2 v1.24.0, R version 3.5 (43). Results were corrected for multiple testing using the Benjamini-Hochberg method, and were filtered for genes with an adjusted *P* value ≤ 0.05 and an absolute fold change ≥ 1.5. Functional enrichment of these differentially expressed genes was performed using an overrepresentation analysis via the R package Gage v2.34.0 (44), testing for enriched pathways from the KEGG database for B. pertussis sp. strain Tohama I. Pathways resulting from this analysis were considered significant based on a false discovery rate (FDR)-corrected *q* value threshold of ≤0.1. To generate counts of mapped reads for each sample and genotype along a defined region of interest, we used IGVtools’ count function v2.4.14 (73).

Volcano plots were generated by plotting log_2_ fold change against −log_10_ of the *P* value for all genes detected in our RNA-Seq analysis. Genome coordinates of putative sRNAs (46) present in the Bordetella genome were added prior to DESeq2 analysis to determine any differential expression among these potential regulatory RNAs. Subsets of differentially expressed genes were generated by filtering locus tags present in other transcriptomic analysis data sets of Bordetella pertussis (21, 28, 30).

### Quantitative PCR.

Wild-type and mutant cells were grown in SS-medium at 37°C. Cultures were harvested at mid-log phase to late log phase and stationary phase. Cell pellets were resuspended in 100 μl Tris-EDTA pH 7.0 with 1 mg/ml lysozyme. RNA was then extracted following the RNAqueous RNA extraction kit protocol (Ambion) and treated for DNA contamination using Turbo DNase I (Ambion). RNA concentrations were measured by nanodrop spectrophotometer and DNA removal was verified by PCR.

To verify gene transcript abundance changes, cDNA was made from 250 ng of each RNA sample using I-script cDNA synthesis kit (Bio-Rad) following kit instructions. All primers used are detailed in Table 2 and were designed to have an annealing temperature of 60°C. Quantitative PCR (qPCR) reactions were set up using SsoAdvanced SYBR green master mix and measured on a Bio-Rad CFX96 instrument. Each sample was run in duplicate with each 10-μl reaction containing 500 nM of each primer pair and 12.5 ng cDNA. The thermal cycling program consisted of an initial melt step of 98°C for 3 min, then 95°C for 15 secs and 60°C for 30 secs for 40 cycles, followed by melt curve analysis. Results were normalized using *rpoB* as a housekeeping gene and fold changes calculated using the threshold cycle (2^-ΔΔCt^) method (74).

### Immunoblotting.

B. pertussis BP338 wild type and endonuclease mutant strains were grown in complete Stainer-Scholte medium to the desired growth phase, mid-log (OD_600_ 0.5 to 0.6), late log (OD_600_ 0.8 to 0.9), and stationary phase (time point = 108 h) or from plates at 72 h. For each time point, 1 ml of culture was harvested by centrifugation and cell pellets were resuspended to an OD_600_ of 5.0 in SS salts (75) to adjust for equal loading. An equal volume of 2× sample buffer was added and the samples heated to 95 to 100°C for 5 min. Each time point sample was run on 12% SDS-polyacrylamide gels and transferred to polyvinylidene difluoride (PVDF) membranes. Membranes were probed with polyclonal antibodies raised against BrkA (76), TcfA (77), and monoclonal antibodies to CyaA (Sigma). Primary antibodies were visualized using horseradish peroxidase (HRP)-conjugated secondary antibodies. The band intensities were then measured using ImageJ (47) to estimate fold changes in protein expression.

### Data availability.

Sequence and count files are available at the Gene Expression Omnibus under accession number GSE164312.

10.1128/mSphere.00650-21.10 TABLE S8 List of operons found to be differentially expressed in both BPrncD45A and BPrneΔCT strains. Download Table S8, XLSX file, 0.02 MB. Copyright © 2021 Ifill et al. 2021 Ifill et al. https://creativecommons.org/licenses/by/4.0/ This content is distributed under the terms of the Creative Commons Attribution 4.0 International license.
