# Supplementary figures and images for: RNase III and RNase E Influence Posttranscriptional Regulatory Networks Involved in Virulence Factor Production, Metabolism, and Regulatory RNA Processing in Bordetella pertussis
Source: mSphere. 2021 Aug 18;6(4):e00650-21. doi: 10.1128/mSphere.00650-21 (PMC8386462; doi:10.1128/mSphere.00650-21)

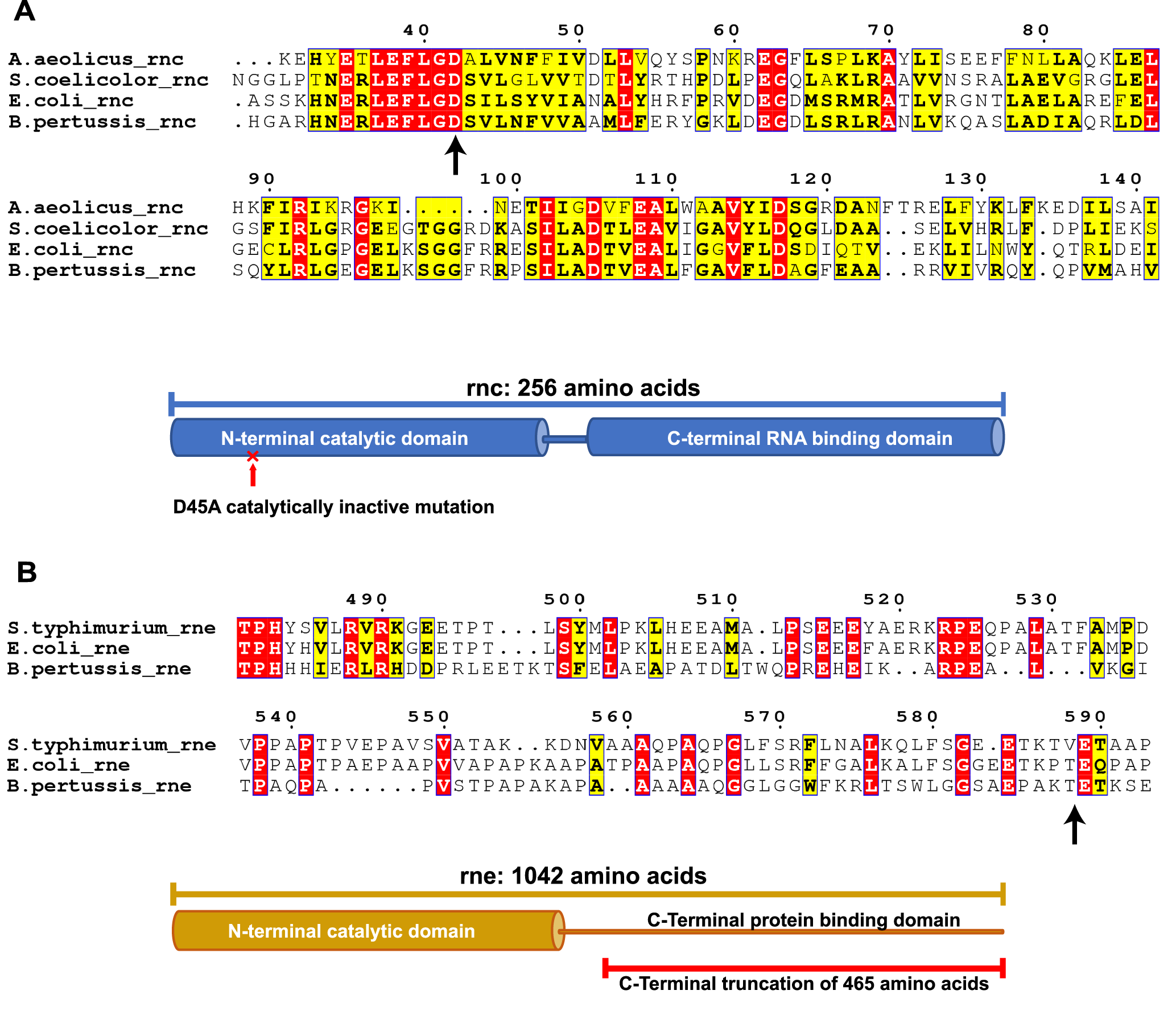

Supplement: FIG S1 [file msphere.00650-21-sf001.tif]

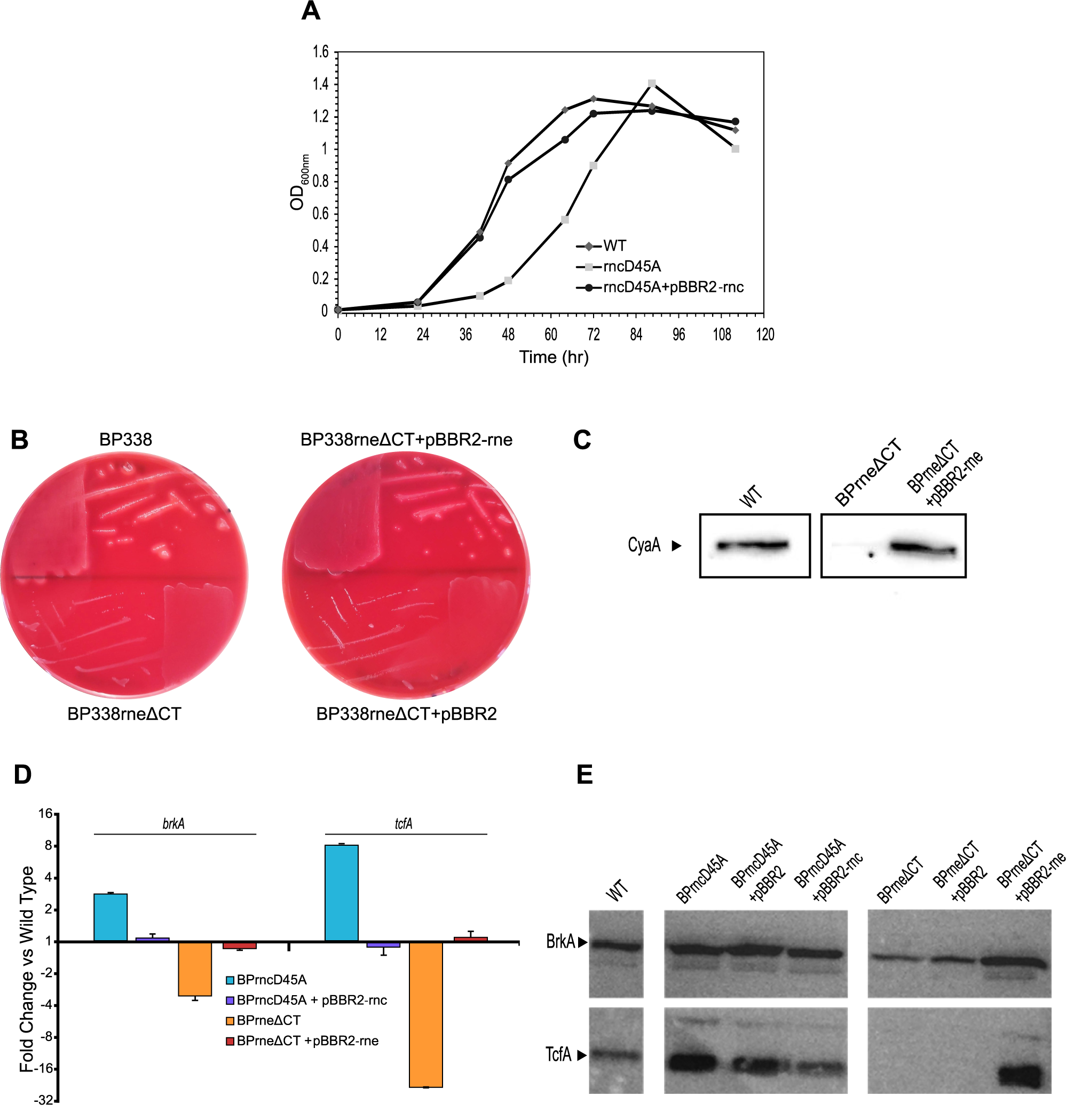

Supplement: FIG S2 [file msphere.00650-21-sf002.tif]
